# Preceding and trailing role-taking in dyad synchronization using finger tapping

**DOI:** 10.1038/s41598-023-36880-0

**Published:** 2023-06-18

**Authors:** Kazuto Kimura, Yuki Tanaka, Taiki Ogata, Yoshihiro Miyake

**Affiliations:** Department of Computer Science, Tokyo Institute of Technology, Yokohama, 266-8502 Japan

**Keywords:** Psychology, Human behaviour

## Abstract

In ensembles, people synchronize the timings of their movements with those of others. Players sometimes take on preceding and trailing roles, whereby one’s beat is either slightly earlier or slightly later than that of another. In this study, we aimed to clarify whether the division of preceding and trailing roles occurs in simple rhythmic coordination among non-musicians. Additionally, we investigated the temporal dependencies between these roles. We conducted a synchronous-continuous tapping task involving pairs of people, whereby pairs of participants first tapped to synchronize with a metronome. After the metronome stopped, the participants synchronized their taps to their partners’ tap timings, which were presented as auditory stimuli. Except in one trial, the pairs involved participants taking on preceding and trailing roles. Compared to the participants taking on the trailing role, those taking on the preceding role demonstrated enhanced phase-correction responses, while those taking on the trailing role significantly adapted their tempos to match those of their partners. As a result, people spontaneously divided into preceding and trailing roles. The preceding participants tended to reduce asynchronies, while the trailing participants tended to match their tempo to their partners’.

## Introduction

In rhythmic contexts, people synchronize the timings of their movements with those of others. For example, in an ensemble, performances are conducted so as to enable the timings of the sounds to match. The performers should match the tempo (beats per minute) and timing of playing to achieve synchronization. In particular, the leader-follower strategy helps match tempos. For instance, in a string quartet, the viola player, as the tempo-follower, matches their tempo with that of the first violin player as the tempo-leader; similarly, the first violin player (as the tempo-follower) matches their tempo with that of the cello player (as the tempo-leader)^1^ though the results were obtained for a particular musical piece performed by a specific quartet. However, players cannot synchronize their sounds just to match tempos. Even if they play their instruments at the same tempo, synchronization can be achieved only if the timing of playing is properly corrected to match that of the other player. However, when a person attempts to play in synchronization with another, their timings do not always match^1–3^. In other words, there are situations in which some performers play a sound first (preceding) and the rest play a sound after a slight delay (trailing). For example, in the study mentioned above of the quartet, the first violin and cello make a sound approximately 10 to 15 ms earlier than the second violin and viola^1^.

In the previous studies, musical performances involved professional players, whereby their experience as professional musicians would affect role-taking. Additionally, many other elements, such as different instruments, parts (melody or harmony), and roles (conductor or player), could affect timing performance and role-taking in musical ensembles^4^. Does the division of role-taking occur in simple rhythmic coordination among non-musicians? If so, what relationship is observed between role-taking for the tempo and timing? Hereafter, the terms “leader” and “follower” are used to denote a tempo-leader and tempo-follower, respectively.

Multiple studies investigating the fundamental features and mechanisms of rhythmic coordination as well as timing control through finger-tapping experiments have been conducted^5,6^. In such studies, the tasks involve people tapping their fingers to achieve synchronization with external stimuli using various devices, such as metronomes. In finger-tapping tasks to achieve synchronization using the metronome as an external stimulus, people tap slightly earlier (approximately several tens of ms) than the metronome’s beat. In other words, the taps tend to precede the corresponding metronome stimuli. This phenomenon is called negative mean asynchrony (NMA)^7,8^. People are sometimes unaware of NMA^9^, yet when the metronome tempo changes, people immediately modify their own intervals to follow the metronome’s^10^.

Rhythmic coordination between people has different characteristics from synchronization with the metronome^11–17^. In some previous studies, researchers used a dyad synchronous-continuous tapping task to investigate the characteristics of rhythmic coordination between people^13,15,16^. First, a metronome with a constant tempo is presented to two participants simultaneously, after which the participants synchronize their finger tapping with the metronome’s beat (synchronous phase). After the metronome stops, the participants continue tapping to maintain the metronome’s tempo while the auditory feedback obtained through the timing of the partner’s taps is presented (continuous phase). Regarding the correction of tempos in the continuous phase, the leader-follower relationship occurs between paired non-musicians^14^. The cross-correlation analysis found a positive correlation of inter-tap intervals (ITIs) at lag -1 between the paired participants. This result showed that compared to followers, leaders were highly likely to maintain their ITIs. Contrarily, followers were highly likely to match their ITIs with the previous ITIs of the leaders.

People synchronize external rhythmic signals using two correction mechanisms: phase- and period-correction. First, researchers have investigated how people modify the timings of their movements to achieve synchronization with rhythmic stimuli, finding that they modify the timing of the next tap in response to the asynchrony between their previous tap and that of the stimulus. They thus adjust the timing of the next tap to reduce the error between the last tap and that of the stimulus. This approach is called phase-correction^18–25^. Second, people adjust the intervals of tapping (or internal timekeeper) to match the intervals of the stimuli involved^10,26^, generally increasing/decreasing the next tap interval if the previous tap interval is shorter/longer than that of the stimulus. This approach is called period-correction. Some studies have demonstrated that a phase-correction model combined with period-correction can be utilized to reconstruct synchronization with a metronome or other people^27–29^. That is, people can decide or correct one tap timing using two different internal mechanisms.

In musical ensembles and simple rhythmic coordination, such as that relying on dyad finger-tapping, common temporal dependencies between tempos and leader-follower relationships have been observed^1,14^. However, in the context of rhythmic coordination, the universal observation of role-division regarding timing, such as preceding and trailing timings, observed in music ensembles has yet to be determined. In other words, the issue of whether role-taking in timing is peculiar to musical ensembles involving professional musicians has yet to be addressed. This study aims to clarify whether non-musicians precede or trail the timing of their partners in simple rhythmic coordination, such as those involving dyad finger-tapping tasks. Furthermore, how the preceding and trailing participants synchronize with their partners is also examined. As mentioned above, the tap timing is controlled by two different mechanisms, which are related to corrections of asynchrony and tempo. First, we estimated the degrees of phase-correction responses of the preceding and trailing participants. Second, we investigated the relationship between the roles regarding timing (preceding and trailing) and tempo (leader and follower). As described above, people take the leader/follower roles in the dyad finger-tapping task^14^. Thus, we investigated the degree to which the preceding and trailing participants matched the tempo of their partner.

To achieve the objectives of this study, we conducted a dyad synchronous and continuous finger-tapping task. The study sample involved in the experiments comprised 12 participants (in six groups) without special musical training or experience. At the beginning of each trial, the participants listened to auditory stimuli obtained from a metronome at intervals of 700 ms, and they performed this task 10 times. The participants were instructed to finger-tap in synchronization with the stimulus from the fifth iteration. After the metronome stopped, the participants were presented with auditory stimuli as their partners tapped. We instructed the participants to continue tapping to maintain the metronome’s tempo in sync with their partners’ auditory stimuli. Instructing the participants to both synchronize with their partners and maintain the tempos resulted in the participants becoming confused because the two instructions sometimes conflicted with each other^30–32^. When the participants found it difficult to both maintain the tempo and synchronize with their partner at the same time, some participants would prioritize maintaining the tempo and others would prioritize synchronization. To avoid such a situation, we instructed the participants to prioritize achieving synchronization with their partners over maintaining the tempo if they felt it difficult to achieve both. We prioritized synchronization because the timing difference between the participants is the focus of this study.

## Results

We recorded the tap times for 100 taps after the metronome stopped for all pairs of participants. In each trial, after the metronome stopped, the data obtained from 20 taps were considered the transition state, and the data obtained from the remaining 80 taps were used for analysis. We analysed the synchronization errors (SEs) between the participants and the ITIs of each participant. As 1 trial was excluded from analysis because the participants failed to conduct the task during the trial 59 trials were analysed. Mean SEs and ITIs were $85.8 \pm 53.13$ ms and $602.4 \pm 61.3$ ms, respectively.

**Figure 1:**
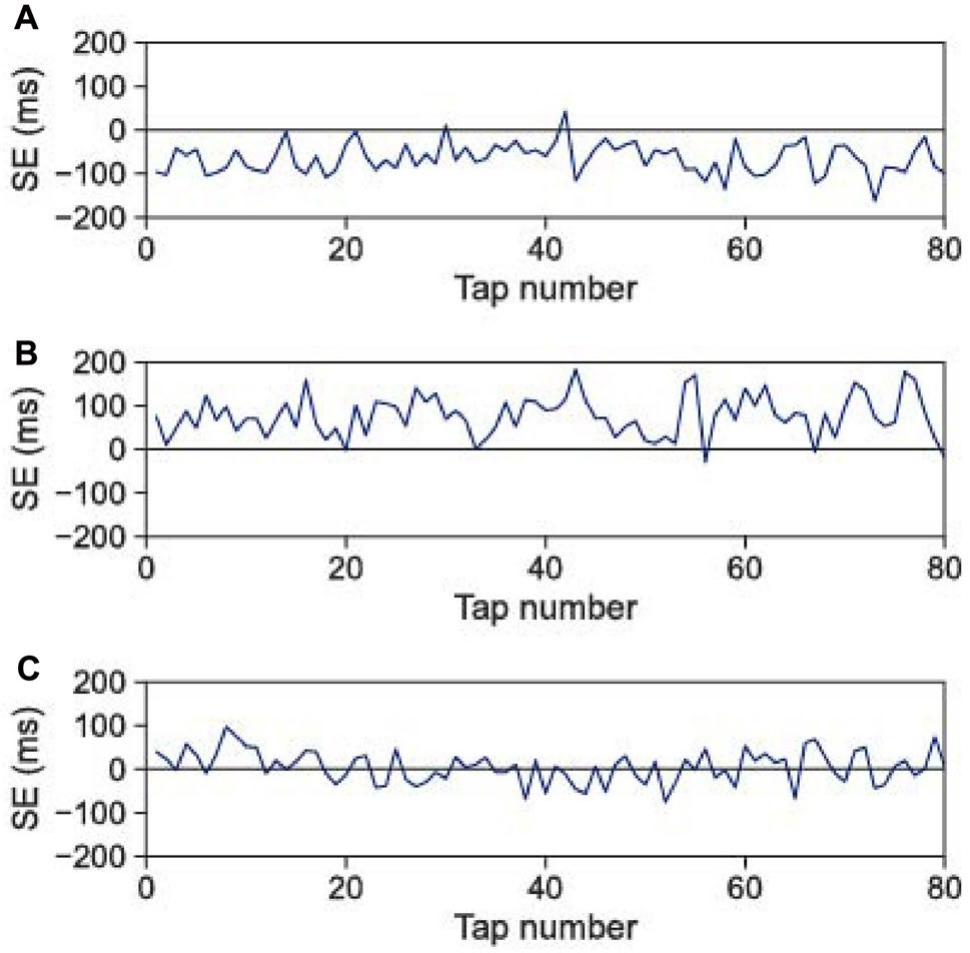
Typical samples of the SE time series. (**A**) Sample in which one participant preceded and the other trailed. The SEs were almost entirely negative throughout the trials. (**B**) Another sample in which participants took on the preceding and trailing roles. The SEs were almost entirely positive throughout the trials, thereby meaning that the preceding and trailing roles among the participants were reversed on the trial (**A**). (**C**) Sample in which the preceding and trailing roles were not observed.

**Figure 2:**
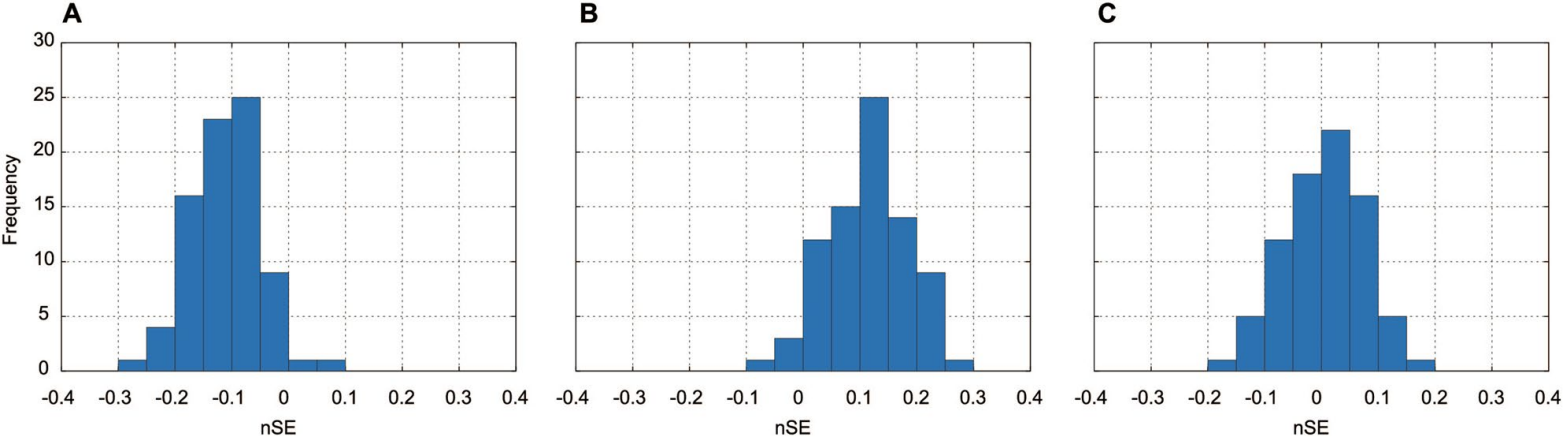
Typical histogram samples for nSEs, which were the SEs divided by the corresponding ITIs of one participant of the pair. (**A**) Sample wherein the nSE was distributed around a negative value. (**B**) Sample wherein the nSE was distributed around a positive value, implying that the preceding and trailing roles of the participants were reversed from trial (**A**). (**C**) Sample wherein the nSE was distributed around 0.

### Preceding-trailing role-taking

Figure 1 illustrates samples of the SEs’ time-series data from the first 20 taps. In Figs. 1A,B, one participant preceded the tap timing throughout almost the entire trial, while in Fig. 1C, a relationship between the preceding and trailing roles was not observed. Figure 2 presents distribution histograms for the normalized SEs (nSEs) of each sample in Fig. 1. Here, the SEs were divided by the corresponding ITIs of one participant in the pair because the SEs were correlated with the ITIs^16^. The nSEs were distributed around a negative value (Fig. 2A), a positive value (Fig. 2B), and 0 (Fig. 2C).

First, for each trial, we performed the Augmented Dickey-Fuller (ADF) test to investigate the stability of the SE time series. The results confirmed that all the SE time series were steady ($p < 0.05$), thereby revealing that the relationships in tap timings were stable throughout all the trials. Next, we investigated whether the participants took on the roles of preceding and trailing. If the participants were divided into the preceding and trailing roles, the averaged SEs would be positive or negative because the SEs were stable throughout the trials. Therefore, under the null hypothesis that the mean nSE value is 0, a *t*-test was performed on the standardized SEs based on the corresponding ITIs. As a result, in almost all the trials, the standardized SE values were significantly different from 0 ($p < 0.05$), except for one trial. Therefore, the participants took on preceding and trailing roles in almost all the trials. For each pair, the numbers of trials in which each participant took on the preceding or trailing roles are listed in Table 1. Among most of the pairs, one participant took on the preceding/trailing role.

**Table 1:** Number of trials in which each participant took on the preceding/trailing role in a pair. We divided each pair into participants A and B depending on their seats.

Pair No.	1	2	3	4	5	6
A preceding/B trailing	0	10	0	3	10	10
B preceding/A trailing	9	0	10	6	0	0
No early-late	1	0	0	0	0	0

### Phase-correction responses

**Figure 3:**
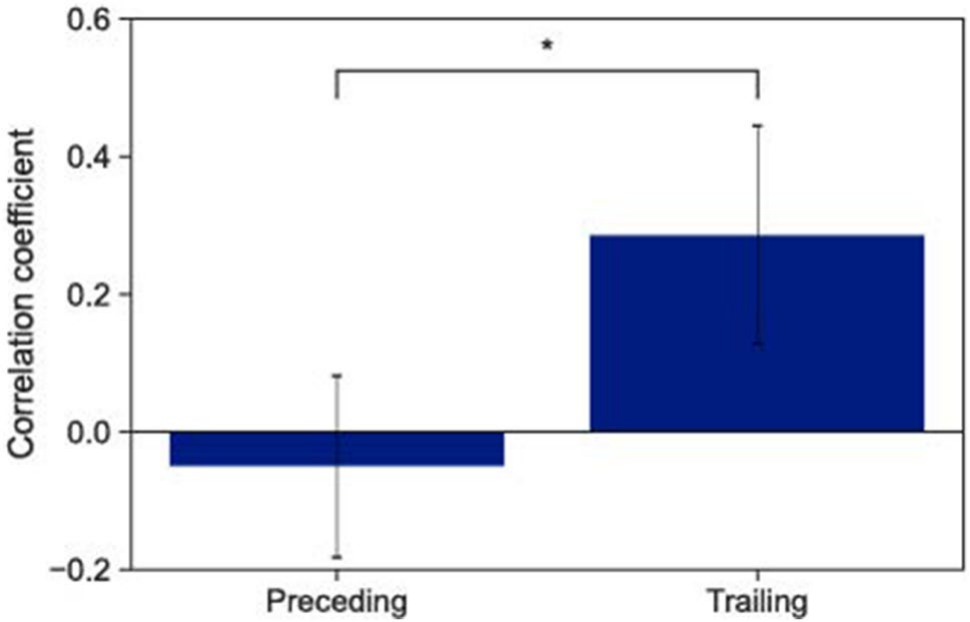
Averaged degree of the phase-correction responses, $\alpha$, among participants who took on the preceding and trailing roles, where the higher the value, the more significant the phase-correction response. PCR = phase correction response. The error bars represent the standard deviations between the participants, and * indicates $p < 0.05$.

We then investigated the timing-control characteristics of the participants who took on preceding and trailing roles. Here, we estimated the phase-correction degree, which reflects one of the tap-timing control mechanisms. In the following analyses, we excluded the trial in which preceding and trailing roles were not observed. The degree of the phase-correction responses was estimated using the phase-period correction model^29^. The phase-correction responses demonstrate the degrees to which the participants corrected the timing of the next tap using the SE value of the previous tap. The degrees of the phase-correction responses, $\alpha$, were estimated using the bounded Generalized Least Squares (bGLS) method proposed by Jacoby et al.^33,34^. Figure 3 shows the averaged degree of phase-correction responses obtained from the participants who took on preceding and trailing roles. To investigate the differences in the phase-correction responses between these participants, we used a first-order linear mixed-effects model, finding that the phase-correction responses of the participants that took on the preceding role were significantly higher than those of the partner taking on the trailing role ($t(114) = 4.14$, $p <0.001$) Therefore, compared to the participants who took on the trailing role, those who took on the preceding role significantly corrected the timing errors.

### Relationship between preceding-trailing and leader-follower role-taking

**Figure 4:**
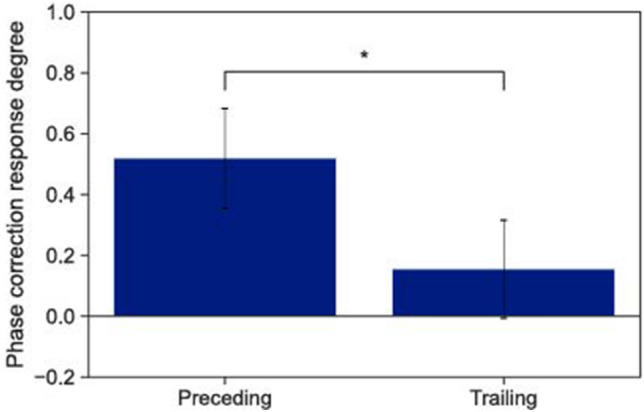
Averaged correlation coefficients of the participants who took on the preceding role versus the trailing role between the participants’ ITIs and the previous ITIs of their partners. Higher values indicate an increased tendency for participants to synchronize their ITIs with the preceding ITIs of their partners. The error bars represent the standard deviations between the participants, and * indicates $p < 0.05$.

Finally, we investigated whether the preceding-trailing role-taking was related to the leader-follower role-taking, which was observed in the dyad finger-tapping task^14^. First, we calculated the windowed cross-correlation between the time-series of the participants’ (n)-th ITI and their partners’ (n-1)-th ITI. The correlation coefficient shows the degree to which the participants synchronized their ITIs to those of their partners’ previous ITIs. In the dyad finger-tapping task, the tempo accelerated gradually^15,16^. Therefore, we calculated the correlation using the window cross-correlation approach, whereby the temporal local dependencies between two time-series were investigated while excluding global tendencies in the time series, such as trends^35^. The higher the value of the positive correlation coefficient, the more significantly the participants synchronized their next ITIs with the previous ITIs of their partners. In other words, a positively high value means that the participant took on the follower role. If the value is closer to 0, the participant took on the leader role. Figure 4 shows the averaged coefficient values of the preceding and trailing roles. A first-order linear mixed-effects model revealed significantly higher levels of correlation among the participants who took on the trailing role than among those taking on the preceding role ($t(114)=6.79$, $p<0.001$). Therefore, the participants who took on the preceding and trailing roles tended to be the tempo leaders and followers, respectively.

## Discussion

In this study, we aimed to clarify whether preceding and trailing roles occur in simple rhythmic coordination. Therefore, we conducted a dyad synchronous and continuous finger-tapping task. As a result, except in one trial, the participants took on preceding and trailing roles. Additionally, different strategies for synchronization between the preceding and trailing participants were observed. The preceding participants showed stronger phase-correction responses than the trailing ones. However, the trailing participants showed stronger period correction than the preceding ones. Thus, in terms of the tempo, the trailing participant followed the preceding participant, who was the tempo leader.

As mentioned in the introduction, preceding and trailing role-taking has been observed in music ensembles^1,36^. In this study, preceding and trailing role-taking was also observed in simple dyadic rhythmic coordination. The participants in the experiments in this study were not professional musicians and had not previously played specific musical roles, such as leader and follower. However, similar to other musical ensembles, the participants tended to divide into preceding and trailing roles. Additionally, the preceding and trailing roles remained stable throughout the trials. Therefore, role-taking is a fundamental feature in dyadic rhythmic coordination.

How did the participants divide into the preceding and trailing roles, even though we instructed them to synchronize with each other? The participants could not have intentionally taken on their roles. In synchronization with the constant-tempo metronome, people are generally unaware of their NMA^9^, although the NMA can sometimes be as long as 100 ms^37^. In our experiment, the average SE was less than 100 ms. Thus, the participants were hardly aware of the asynchrony and preceding/trailing roles. Individual characteristics of the paired participants would result in the division of preceding and trailing roles. In many pairs, one participant (almost) always preceded/trailed the timing of their partner (Table 1). One possible reason behind the individual characteristics is the tendency for people to tap according to external rhythmic stimuli such as the metronome in advance^7,8^. The degree of this negative asynchrony is known to vary from person to person^37,38^. In dyadic rhythmic coordination, people tap to respond to each other before experiencing the stimuli of their partners. In this task, people with a tendency to tap more than their partners before the external stimuli would tap earlier than their partners. Therefore, participants would divide spontaneously into the preceding and trailing roles, and the roles could remain stable in and between trials for one pair. As the synchronization phase was too short in our experiment, we could not measure the degree of the NMA of each participant between the metronome stimuli and their taps. In the future, the relationship between the degree of the NMA and preceding/trailing roles should be determined.

Another possible reason for this phenomenon is individual differences in personal tempos^39,40^. A personal tempo is favourable for individuals when they naturally produced rhythms, such as walking and cycling. The interpersonal variability of personal tempos is significantly large^41^, and the variability within the individual is significantly small^42,43^; indeed, personal tempo shows consistency across days^43,44^. Mean asynchrony in a piano duo is correlated with the mean tempo in the solo task^3^. Additionally, the faster participant in solo tasks tends to precede the timing of the slower one. In our experiment, the tempo (the averaged ITIs) could be unknowingly guided to the participant’s personal tempo, thereby resulting in a difference in the tempo of the taps. Consequently, the participants would spontaneously take on preceding and trailing roles.

In the dyad synchronous and continuous tapping task, the tempo of one participant influenced the tempo of the other^15^. In this study, the tempo change was investigated in a solo synchronous and continuous task, wherein the participants tapped without a stimulus in the continuous phase to maintain the metronome tempo in the synchronous phase. In the dyad task, the tempo of the two participants was accelerated; however, one participant in the pair accelerated the tempo in the solo task more than the other, and the acceleration of the tempo was observed in our experiments. Note the ITI average was less than the metronome tempo (700 ms), and the participants who accelerated the tempo more than their partners thus took on the preceding role. Additionally, this mechanism could explain why the trailing participants showed higher correlation coefficients between their ITIs than the preceding ones (Fig. 4). The trailing participants would ensure that their tempo followed that of the preceding ones, who changed their tempo more strongly. Contrariwise, the preceding participants tried to synchronize with their partners using a stronger phase correction than the trailing ones did (Fig. 3), rather than adapting their tempo to the partner.

We experimented with only one metronome tempo (700 ms). The tempo of the metronome in the dyad synchronous and continuous task affects the strategies for synchronization with the partner^16^. For example, people were affected more strongly by the previous ITIs of the partner at slow tempos than fast ones. Thus, the role-taking for the timing (preceding/trailing) and their synchronization strategies changed along with the tempo. Moreover, the role-taking and relevant strategies changed when the tempos of the internal timekeepers were different between the paired participants. If metronomes with different tempos are presented to each participant in the synchronous phase, the participants could start the continuous phase with different tempos of their internal timekeepers. This task can enhance the division of the role-taking between the paired participants and reveal the mechanism of the division more clearly.

In this study, leader-follower role-taking in tempos was observed between the participants as they took on preceding and trailing roles. However, as demonstrated in a previous study^13^, the hyper-follower relationship, and not the leader-follower relationship, was observed between participants. In other words, both participants synchronized their ITIs to the previous ITIs of their partners. This difference in tempo dependency is likely caused by the instruction differences between the current and previous studies. In previous studies, participants were asked to both maintain the tempo and synchronize with the auditory stimulus of the partner. However, in this study, the participants were instructed to prioritize synchronization with their partner over tempo maintenance. Thus, in previous studies, participants strongly attempted to maintain the metronome tempo, unlike the current study, and this is possibly related to the emergence of hyper-follower relationships in previous studies. The effect of the instruction on the preceding/trailing roles should also be investigated in future research.

In the context of tempo dependency, the participants taking on the preceding or trailing roles were the leaders or followers, respectively. Timer *et al.* found similar role-taking characteristics in the timing and tempo in one string quartet^1^, where the first violin player preceded the timing of the viola player, and as a result, the first violin led the tempo of the viola. However, various relationships in timing and tempo have been observed in musical ensembles^1–3^. For example, even in the string quartet mentioned above^1^, the cello preceded the second violin, but they were hyper-followers of each other with regard to tempo dependency. Additionally, in the context of timing, although there was no preceding-trailing relationship between the first violin and the cello, the cello was the leader in the tempo. Therefore, the performers would intentionally adjust the relationships observed in the timing and tempo according to their musical roles while overcoming the individual features mentioned above.

In simple dyadic rhythmic cooperation, the participants divided their roles into preceding and trailing ones. The role-taking was stable throughout the trials; however, the synchronization strategy was different between the preceding and trailing participants, such that the preceding participants showed stronger phase-correction responses than the trailing ones. By contrast, the trailing participants corrected their periods to match their partners’. Hence, the preceding and trailing participants were the tempo leaders and followers, respectively.

## Methods

### Participants

A total of 12 people (six pairs) participated in this study (10 male and 2 female, aged 22-32 years). All the participants were right-handed with no hearing or visual impairments, and they had no training in professional music. The paired participants were acquainted. The study was conducted in accordance with the Declaration of Helsinki and approved by the Ethical Committee of the Tokyo Institute of Technology. Written informed consent was obtained from each participant.

### Materials and stimuli

We used pressure sensors (FSR-406, Interlink Electronics, US) to record the participants’ taps. A microcomputer (Arduino Mega 2560 Rev3, Arduino, US) was used to record the tap timing and control the auditory signals. As the auditory stimulus, a sound of 500 Hz was presented for 100 ms via earphones (SHE3010WT, PHILIPS, Nederland). The earphones and pressure sensors were directly connected to the microcomputer. The time resolution to control the stimuli timing and to record the tap timing was less than one millisecond. Moreover, white noise was presented to each participant to mask external noise via headphones (HPH-50B, YAMAHA, Japan) connected to a different personal computer from the one controlling the microcomputer during the trials. White noise was presented to each participant via headphones (HPH-50B, YAMAHA, Japan) during the trials.

### Task

The task consisted of dyad synchronous and continuous tapping task. First, both participants heard 10 auditory stimuli from the metronome during the synchronization task. The metronome tempo was 700 ms. From the fifth stimulus, the participants began to synchronize their tapping with the metronome’s stimuli. After the 10th metronome stimulus was completed, the task moved to the continuation part of the task, where the auditory stimulus was presented at the timing when the partner tapped. The participants were asked to synchronize their taps with the stimulus from the partner and maintain the metronome’s tempo. We instructed the participants to prioritize synchronization with their partner over tempo maintenance. One trial of the tapping task was completed when both participants tapped 100 times in the continuous part of the task.

### Procedures

The participants in the pair sat in seats placed back to back. The participants were randomly assigned one of the two seats and wore eye masks, earphones, and headphones throughout the trials. Before the experiment, the volumes of the auditory stimuli and white noise were adjusted and remained fixed throughout the experiment.

The trials were conducted 10 times for each pair. Before conducting the experiments, the participants performed some practical trials. To minimize the effect of participant fatigue, we took at least 1-minute breaks between each trial and 5-minute breaks after every 5–6 trials. In addition, the participants could take additional breaks at any time if they wanted. The experiment took approximately 1 h and 30 min for each pair.

### Statistics

We excluded one trial from the analysis because the ITIs gradually increased throughout the trials. In dyadic synchronization tasks, the ITIs usually decrease throughout a trial^15^. Therefore, we determined that the participants did not understand the instruction correctly and failed to synchronize with each other during the trial. The data obtained from the final 80 taps in the synchronization task were used for the analysis. All analyses were performed using MATLAB (MATLAB 2022a, The MathWorks). The ITIs, SEs, and normalized SEs (nSE) were determined as follows:

1 $$\begin{aligned}&ITI_i (n)=Tap_i (n) - Tap_i (n-1) \end{aligned}$$

2 $$\begin{aligned}&SE_{ij} (n)=Tap_i (n) - Tap_j (n) \end{aligned}$$

3 $$\begin{aligned}&n SE_{ij} (n)=SE_{ij} (n) / ITI_i (n), \end{aligned}$$

where $(i, j) = (A, B) or (B, A)$, with A and B indicating the two participants in a pair.

The values of the SEs were correlated with the corresponding ITIs^16^. Thus, we utilized nSEs in this analysis. We also performed an ADF test for the nSEs of each trial for each pair of participants to investigate the stability of the nSE time series. The significance level was set at $\alpha$ = 0.05. To investigate whether the averaged nSE was zero, we conducted a *t*-test for the nSE of each trial with the Holm-Bonferroni method for multiple comparisons, and set the significance level $\alpha$ at 0.05.

To estimate the degree of phase-correction responses, we used a phase-period correction model^29^ and the bGLS method^33,34^. The phase-period correction model was determined as follows:

4 $$SE_{ij} ( k+1)=(1-\alpha ) SE_{ij} (k)+t(k)+T' (k)+M(k+1)-M(k)-ITI_j (k+1)$$

5 $$\begin{aligned}&=(1-\alpha ) SE_{ij} (k)+t(k)+H(k)-ITI_j (k+1) \end{aligned}$$

6 $$\begin{aligned}&t(k)=t(k-1)-\beta SE_{ij} (k), \end{aligned}$$

where the parameters $\alpha$ and $\beta$ indicate the degrees of phase and period corrections, respectively. *M* and *T* represent the motor noises and noises of the internal timekeeper, respectively, and *t* indicates those of the internal timekeeper. To calculate the degree of phase-correction responses, we estimated $\alpha$ for each trial using Jacoby’s method^33,34^.

We tested the effects of the preceding and following roles on the phase-correction response while considering the effects of participants and pairs using the generalized linear mixed model^45^. The significance level $\alpha$ was set at 0.05.

7 $$\begin{aligned} (Parameter)=(Role)+(1|Participant)+(1|Pair) \end{aligned}$$

The variable Parameter represents the value of the parameters, alpha or beta. The variable Role takes the value +1 if the participant’s role was preceding tapper and -1 if the participant’s role was trailing tapper, and the variables Participant and Pair represent the differences between participants and pairs, respectively.

To investigate tempo dependency between the participants, we calculated the windowed cross-correlation of the ITIs between the participants^35^. In the calculation of the cross-correlation, this method can suppress the influence of trends over the entire time series and extract only local influences. The mean coefficient at lag -1 represents the degree to which participants synchronized their ITIs to the previous ITIs of their partners. We set a window of six taps and calculated the correlation coefficient between the (n)-th ITI of the participant and the (n-1)-th ITI of the partner in the windows. Next, we shifted the range of the window range by one tap and obtained the average value of the correlation coefficient in the trial. The closer the obtained mean value was to +1, the more likely it was that the participants modified their ITIs depending on the previous ITIs of their partners who acted as followers. The closer the value was to 0, the higher the tendency was of the participants’ ITIs to be independent of the previous ITIs of their partners who acted as leaders. We tested the effect of the preceding and trailing roles on the leader-follower relationship using a generalized linear mixed model^45^. The significance level $\alpha$ was set at 0.05.

8 $$\begin{aligned} (Correlation Coefficient)=(Role)+(1|Participant)+(1|Pair) \end{aligned}$$

## Data Availability

The datasets used and/or analysed during the current study available from the corresponding author on reasonable request.
